# Health-Aware Model-Predictive Control of a Cooperative AGV-Based Production System

**DOI:** 10.3390/s19030532

**Published:** 2019-01-27

**Authors:** Beata Mrugalska, Ralf Stetter

**Affiliations:** 1 Faculty of Engineering Management, Poznan University of Technology, 60-965 Poznan, Poland; 2 Faculty of Mechanical Engineering, University of Applied Sciences Ravensburg-Weingarten, 88250 Weingarten, Germany; stetter@hs-weingarten.de; 3 Steinbeis Transfer Center Automotive Systems, 88212 Ravensburg, Germany

**Keywords:** assembly systems, scheduling, state-of-health assessment, robot control, model predictive control

## Abstract

In the paper, a new scheduling strategy for assembly systems consisting of cooperating Automated Guided Vehicles (AGVs) based on their remaining operational time is developed. The operational time is associated with state of charge and state of health of the AGV battery. While the latter is defined as a possible number of repetitions of a set of given tasks, both are impossible to measure on-line directly with conventional sensors. Therefore, a novel state-of-charge estimator is proposed, which uses battery current and voltage sensor readings. In contrast to the approaches presented in the literature, a comprehensive analysis of its convergence is provided. Subsequently, a state-of-health predictor is developed. With the above measures, a new control strategy for cooperative AGVs is proposed. It is achieved by the allocation of alternative tasks among two cooperating robots referring to the state of the accomplished tasks from the previous stage of the assembly process. The proposed method allows a predictive control of assembly processes with several constraints, e.g., productivity of each assembly station, speed of the communication, or operation capability of the robots involved in the assembling process. The final part of the paper shows an experimental study exhibiting the performance of the proposed approach.

## 1. Introduction

Automated Guided Vehicles (AGVs) in manufacturing and assembly systems are usually battery driven. This fact can lead to several limitations concerning their efficiency, effectiveness, availability, and reliability. For instance, if an AGV needs a long time for battery charging, another AGV must be available to allow continuous operation of the assembly system during the charging period. Even more critically, AGVs with a fading battery capacity might even run out of energy during their operation, causing traffic jams or even the necessity to temporarily stop the assembly process. Therefore, new solutions, where science could provide trustworthy estimations of the state of a battery and an assembly system could react to these estimations, are highly desirable for the production industry. A battery exhibits two main characteristics: the State of Charge (SOC) and the State of Health (SOH) [1,2]. The SOC can be relatively easily monitored if the energy taken from the battery since the last full charge is observed. Still, a full charge can be not always assumed and the manner in which the energy is taken (over a long period of time or abruptly) can also influence the actual SOC. Consequently, the estimation of the SOC is not trivial [3]. The estimation of the SOH is even more complicated [4,5,6]. During its life and its usage, the useful capacity of a battery will decrease because of complex electrochemical processes. If one would like to know the exact remaining useful capacity of its battery, it would be necessary to perform a complete, controlled discharge cycle. In the usual operating environment of the AGV this is just not feasible. Therefore, it is very important to be able to estimate the remaining useful capacity of batteries in the AGVs. However, for the purpose of further deliberations, the definition of SOH is tailored for the AGV performing alternating sets of tasks, e.g., driving forward and backward from and to given places:

- The SOH is a number of cycles $k_{f}$ from a given SOC down to zero.

This definition is very intuitive as it translates directly to the battery SOH. Indeed, if we imagine two batteries with different SOHs, then it goes without saying that the smaller SOH, the smaller $k_{f}$. Moreover, one can also imagine that for each battery $k_{f}$ will decrease over time. This is caused by battery degradation related to the charge/discharge process. Thus, $k_{f}$ can be also used as a measure for predicting AGV-battery Remaining Useful Life (RUL) [7,8,9]. However, this is beyond the scope of this paper.

The control of complex processes with AGVs has been a strongly researched topic for many years [10,11,12]. The elaborated control systems can allow optimization of the effectiveness and efficiency of the transporting AGVs. However, until now, no predictive control framework has been available that would allow inclusion of an estimation of the states of an AGV battery and a prognosis of the associated SOC. Consequently, the main research questions could be formulated as:

- How to design a framework allowing trustworthy estimation of SOC and SOH of AGV batteries along with incorporation of this knowledge within the predictive control strategy of the assembly system?

**Figure 1:**
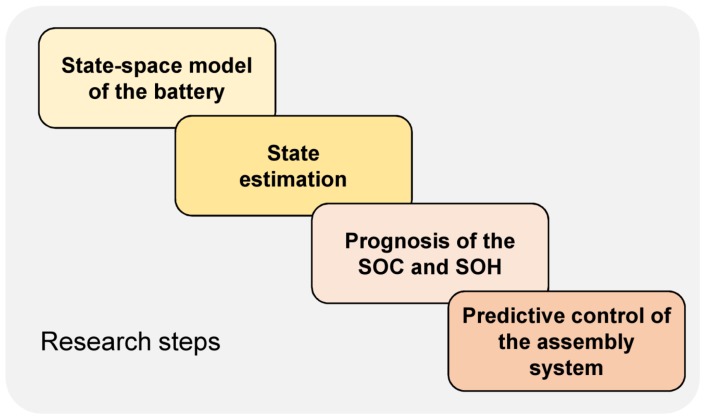
Research steps.

The central approach of this paper can be characterized as follows (c.f. Figure 1):

- Firstly, a state-space model of a battery is derived from the literature.
- Secondly, algorithms, which allow estimation of AGV-battery SOC based on current and voltage sensor measurements, are developed.
- Thirdly, algorithms, which allow the prognosis of the SOH based on the evolution of SOC, are developed.
- Fourthly, control-scheduling algorithms, which allow optimal distribution of workload between two cooperating AGVs, taking into account their SOH, are developed.

The paper is organized as follows: Section 2 gives a short background of the state of the art in the fields of prediction of SOH, models of battery systems, and predictive control of assembly systems. In Section 3, the sample application is explained. Section 4 concentrates on the explanation of the algorithm for state estimation. In Section 5, the algorithm for the prognosis of the SOH is proposed, and evaluated in Section 6. Section 7 focuses on the framework for predictive control of cooperative AGVs. The results of the verification of the earlier research components are described in Section 8. The paper is summarized in Section 9 and a research outlook is given.

## 2. State of the Art

### 2.1. Prognosis of the SOH of Technical Systems

Prognosis means predicting the health condition of a technical system and its components [13,14,15] using knowledge of operations in the past, the current state, and possible and probable future conditions. In many areas of industry, and especially in the control of assembly systems, it is extremely important to predict the time until a failure will occur, which usually leads to an unplanned shutdown of the system. A large amount of research contributes to the prediction of the Estimated Time to Failure (ETF), or, synonymously, RUL. The essence of this endeavor is to detect whether a system, such as a battery, already exhibits serious indicators for and to predict the time within which the system can still be operational below a certain failure threshold. In this context, the term “failure” can be understood as permanent interruptions of the ability of a technical system to perform a required function or task under specified operating conditions [16,17]. This term indicates a complete breakdown of a system, whereas the term “fault” (an unpermitted deviation of at least one characteristic property or parameter of the system from the acceptable, usual, standard condition) only indicates a malfunction, and not a catastrophic event. The possibility of failure must be clarified during the clarification of the task and must be tracked during the whole project by means of requirement engineering (compare [18]). A conscious and computer-based exploration of the functions of technical systems (e.g., [19]) can also help to explore the possibilities for failure and to define the main events which can cause the end of the useful life.

**Figure 2:**
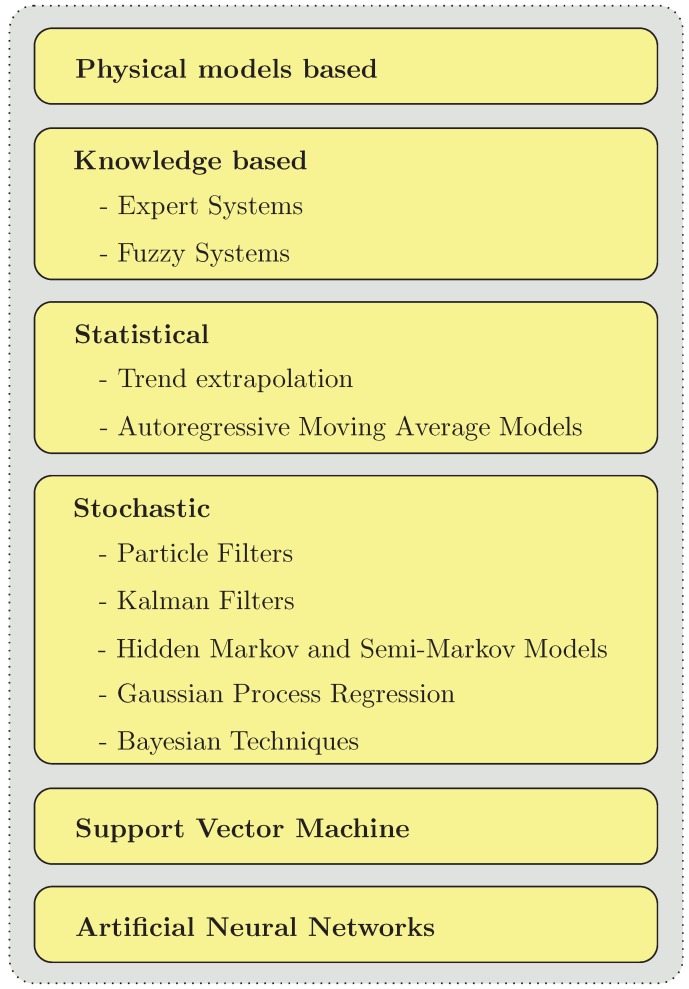
Overview of RUL estimation approaches.

Meanwhile, several excellent reviews summarizing and organizing the respective research are available [20,21,22,23,24,25]. Essential in the direct prognosis of the RUL is the distinction between: physical models-based, knowledge-based, statistical, stochastic, Support Vector Machine (SVM) and Artificial Neural Networks (ANNs) (c.f. Figure 2). Physical model-based approaches use mathematical representations of physical relationships describing a system. They use knowledge of the life cycle of a technical system, knowledge about load conditions during its operation, its geometry, and material properties to identify potential failure mechanisms and to estimate the system RUL [26,27]. Knowledge-based methods rely on the system RUL deduction based on the comparison of an observed system behavior with previously defined failures. Among knowledge-based methods, expert systems and fuzzy systems can be distinguished [28,29]. Statistical approaches estimate the system RUL based on previous inspection results on similar systems based on data such as condition or process-monitoring outputs. Among such methods, trend extrapolation and Autoregressive Moving Average (ARMA) models are the most distinguished in the literature [30,31,32]. Stochastic methods allow determination of the RUL of each component of the system with respect to the expected risk of deterioration under known operating conditions [21]. Among them, Particle Filtering [33], Extended Kalman Filters (EKF) [34], Interval Observers (IO) [35], Unscented Kalman Filter (UKF) [36], Bayesian techniques [37,38], Gaussian Process Regression (GPR) [39] and Hidden Markov (HM) and Hidden Semi-Markov Models (HS-MM) [40] can be distinguished. The Support Vector Machine (SVM) is a machine-learning algorithm which can be applied for nonlinear regression estimation and time series forecasting in the system RUL prognosis tasks [25,41]. Artificial Neural Networks (ANNs) are especially attractive in the system RUL estimation tasks when the mathematical model of the system is not available or when simultaneous system parameters degradation occurs. Such techniques can be relatively easily applied for RUL estimation of complex nonlinear dynamic systems [42,43].

**Figure 3:**
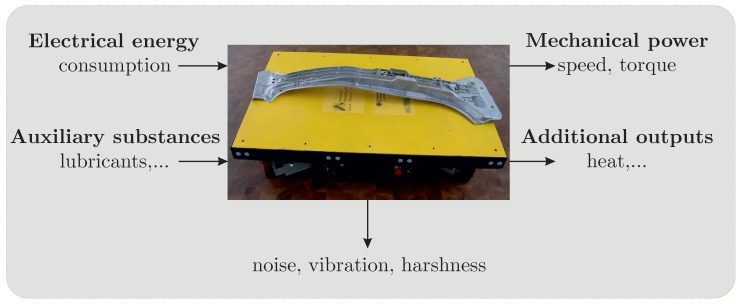
Black Box Diagram AGV.

Several above-mentioned techniques belong to the so-called data-driven approaches. In such case, appropriate data sets allowing for the system RUL estimation are required. To solve this problem, black box analysis can be applied. Figure 3 shows an example of a block box diagram for an AGV.

In this technique, only the inputs and outputs of a system are analyzed. One of the main indicators of degradation of a technical system is the performance of the respective system, which means the quantitative fulfillment of the central functions of the system. For the AGV performance, it is primarily shown by the speed and the torque of the motors. Prominent possibilities for degradation indicators are also the consumption of technical systems, as many forms of degradation result in increased energy consumption. All measurements concerning vibrations are also very important. Secondary indicators could be additional outputs such as the generation of excessive heat or the presence of lubricants at certain unindented places. Current research efforts use performance-deteriorated parameter data and life-cycle data to estimate the RUL [25].

### 2.2. Models of Battery Ageing

Currently, the prevailing battery technology is lithium-ion because in comparison to lead–acid, nickel–cadmium, and nickel–metal–hydride cells, the energy density and durability is rather high [44,45]. However, for an advantageous application of these batteries, knowledge of the degradation behavior is necessary. Several research initiatives concern the investigation and modeling of the battery ageing. Research initiatives apply several algorithms and frameworks such as Bayesian frameworks [46], the Dempster–Shafer Theory (DST) with the Bayesian Monte Carlo (BMC) method [47], EKF [6,48], a dual filter consisting of an interaction of a standard Kalman Filter (KF) and an Unscented Kalman Filter (UKF) [4], Linear Parameter-Varying (LPV) models [49] and nonlinear predictive filters [50]. Some approaches also apply machine-learning methods such as SVM and Relevance Vector Machines (RVM) [5,51], Fractional-Order Techniques (FOT) [52,53], Gaussian Process Regression (GPR) [54]. The concise reviews of estimations for automotive applications are given by Barre et al. [55], Rezvanizaniani et al. [56] and Berecibar et al. [57].

For the estimation of the AGV-battery SOC and SOH, two ageing mechanisms can be distinguished: calendar ageing and cycle ageing. Calendar ageing means a reduction of the battery capacity over time. Unfortunately, this is not a linear process and it is strongly influenced by parameters such as temperature. Cycle ageing occurs during charge and discharge. This process is also influenced by many parameters such as temperature and the discharge and charge characteristics. Both ageing mechanisms lead to resistance growth and capacity fade. A mathematical model for batteries was developed based on an elaborate literature analysis by Taborelli and Onori [58]. They present the following state-space formulation in discrete time for an entity of four cells which they refer to as “average cell”:

(1) $$\left\{\begin{array}{c} SOC\left(l+1\right)=SOC\left(l\right)-\frac{\Delta t}{Q_{nom}}I\left(l\right),\\ V_{CT}\left(l+1\right)=e^{-\frac{\Delta t}{\tau_{CT}}}V_{CT}\left(k\right)+R_{CT}\left(1-e^{-\frac{\Delta t}{\tau_{CT}}}\right)I\left(l\right),\\ V_{Dif}\left(l+1\right)=e^{-\frac{\Delta t}{\tau_{Dif}}}V_{Dif}\left(k\right)+R_{Dif}\left(1-e^{-\frac{\Delta t}{\tau_{Dif}}}\right)I\left(l\right). \end{array}\right.$$

where $Q_{nom}$ stands for the nominal capacity, $V_{CT}$ denotes the charge transfer voltage, $R_{CT}$ represents the charge transfer resistance, $V_{Dif}$ stands for the diffusion voltage and $R_{Dif}$ represents the diffusion resistance. Obviously, in this model the dynamic response of battery cells is modeled using two RC branches ($R_{CT},C_{CT}$ and $R_{Dif},C_{Dif}$) with the respective time constants $\tau_{CT}=R_{CT}C_{CT}$ and $\tau_{Dif}=R_{Dif}C_{Dif}$ which represent charge transfer (CT) and diffusion (Dif) processes in the cells of the battery. While I(l) stands for the measured battery current, *l* is a discrete time, Δt denotes the sampling time. Finally, $Q_{nom}$ represents the battery nominal capacity.

The measured output voltage V(l) is related to the relevant voltage drops:

(2) $$V\left(l\right)=V_{OCV}\left(SOC\left(l\right)\right)-V_{CT}\left(l\right)-V_{Dif}\left(l\right)-R_{0}I\left(l\right),$$

with the average cell open circuit voltage function $V_{OCV}\left(SOC\right)$ and the internal resistance of the battery cell $R_{0}$. Taborelli and Onori [58] define the state vector as $x_{l}={\left[SOC\left(l\right)\;V_{CT}\left(l\right)\;V_{Dif}\left(l\right)\right]}^{T}$. The input of the model is the current $u_{l}=I\left(l\right)$ and its output is the voltage $y_{l}=V\left(l\right)$. This leads to the nonlinear state-space model of the battery:

(3) $$\begin{array}{cc} x_{l+1} & =Ax_{l}+Bu_{k}, \end{array}$$

(4) $$\begin{array}{cc} y_{l} & =g(x_{l},u_{l}). \end{array}$$

The respective matrices *A* and *B* are:

(5) $$\begin{array}{cc} A & =\left[\begin{array}{ccc} 1 & 0 & 0\\ 0 & e^{-\frac{\Delta t}{\tau_{CT}}} & 0\\ 0 & 0 & e^{-\frac{\Delta t}{\tau_{Dif}}} \end{array}\right],\\ B & =\left[\begin{array}{c} -\frac{\Delta t}{Q_{nom}}\\ R_{CT}\left(1-e^{-\frac{\Delta t}{\tau_{CT}}}\right)\\ R_{Dif}\left(1-e^{-\frac{\Delta t}{\tau_{Dif}}}\right) \end{array}\right]. \end{array}$$

It should be noted that the model (3) is linear only with respect to state. The nonlinear output function g(·,·) described by Equation (4) causes the necessity of using nonlinear estimators such as EKF [34,48] to estimate the battery state and provide a novel approach for predicting its SOC and SOH. Models (3) and (4) is further detailed by (7) and (8) along with a novel estimator structure, which overcomes the nonlinearity problem and takes into account inevitable disturbances/noise. Unlike the EKF, its convergence is proven by Theorem 2 in Section 4.

### 2.3. Predictive Control of Assembly Systems

As stated above, an advantageous design of future seating assembly systems will use AGVs for transportation purposes. Important objectives are to provide a maximal flexibility for additional seating variants, and to ensure production reliability. It will require appropriate process control strategies. These strategies should allow tolerance of some level of faults in the processes. In recent years, an active fault-tolerant predictive-control framework was developed [59,60], which is based on a description of the assembly system as a Discrete-Event System (DES) [61,62] and applies the Discrete-Event Interval Max-Plus Algebra (DEIA-PA) [63,64].

Based on this mathematical description, a Model-Predictive Control (MPC) is to be developed which overcomes the main drawback of the above approaches pertaining to a concurrency issue. Indeed, the class of systems, which can be tackled with the above-mentioned approaches, excludes the possibility of a concurrency. It means that they cannot be used for controlling cooperating AGVs capable of realizing alternative tasks or sets of tasks. The objective of the subsequent section is to provide a description of the seat-assembly system along with its outlet layer composed of two cooperating AGVs.

## 3. Seat-Assembly System

**Figure 4:**
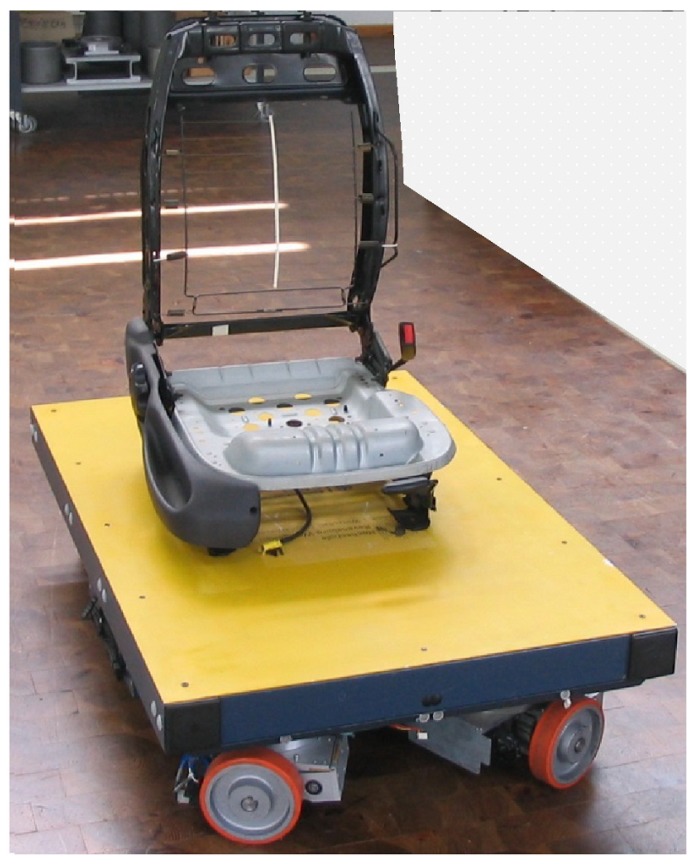
AGV in action: transport of a seat frame.

Contemporary manufacturing and assembly systems in the automotive industry must be adapted for agile production requirements, because of unpredictable product demand and rapid product launches [65,66]. The requirements also apply to automotive seat-assembly systems. It is required to apply flexible solutions without large investments which can be rather quickly implemented. To achieve this goal, AGVs can be used to deliver components to the assembly system (compare [67]). This type of assembly system is usually located close to the car production line and delivers seats just in sequence to the car assembly location (compare [68]). The seats cannot be produced in advance and stored close to the produced cars due to limited space and a huge variety of types of seats. In the seat-assembly system, the different components such as frame, foams, trim, headrest, comfort systems, safety systems, and various covers, which are usually produced at other locations, are assembled. It is usually sensible to divide the assembly of the components such as frame with mechanical and electrical components from the assembly of foam, trim, and covers, as the personnel qualification and the cleanliness requirements are different. The paper focuses on the seat-assembly system and in particular on the transportation problem between two buildings with the application of the AGV presented in Figure 4.

**Figure 5:**
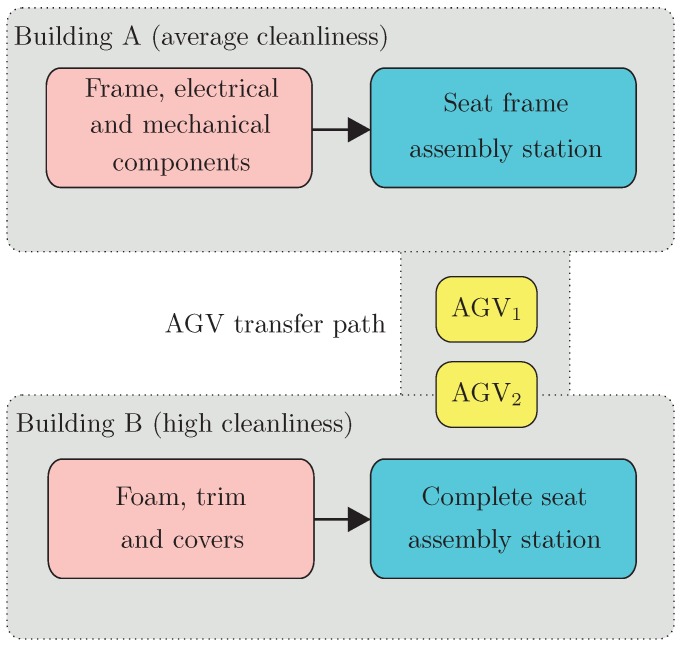
Seats assembling system.

The AGV shown in Figure 4 is based on a unique design developed at the University of Applied Sciences Ravensburg-Weingarten (compare [69]). This AGV concept disposes of certain qualities which are important for the application in the seating assembly plant. Most noteworthy are the unlimited maneuvering possibilities that result from the unique steering concept [70]. It consists of four driving modules which can be oriented by means of balancing the torque of the two drive motors at the two wheels of each drive module. This concept enables the AGV to drive in any direction without any prior maneuvers. This leads to an efficient use of floor space and increases the agility level. Consequently, AGVs that are based on this steering concept are suited to addressing the transportation problems in the seating assembly plant. The investigated problem boils down to the transportation of the seat frame with all mechanical and electrical components such as airbag, belt retractor, and belt lock from the seat frame assembly station located in the Building A, where these components were fitted, to the Complete Seat-Assembly Station located in the Building B, where the foam, trim, and covers will be added (c.f. Figure 5). Between the buildings there is a pathway for AGVs. In the sample scenario two AGVs called AGV 1 and AGV 2 could perform the transportation tasks. Please note that these two cooperating AGVs introduce concurrency into the overall production system.

**Figure 6:**
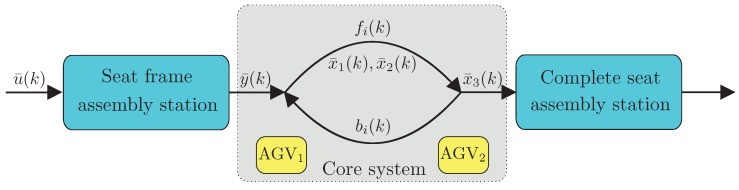
Sample transportation scenario.

The above-described transportation scenario, which is a sequential process, can be depicted in the form of the graph presented in Figure 6.

Where:

- *k*—is the event counter associated with the realization of *k* seat frame transportation,
- ${\bar{x}}_{1}\left(k\right)$—is the start time of the first AGV for *k*-th event counter,
- ${\bar{x}}_{2}\left(k\right)$—is the start time of the second AGV for *k*-th event counter,
- ${\bar{x}}_{3}\left(k\right)$—is the seat frame arrival time *k*-th event counter,
- $\bar{y}\left(k\right)$—is the delivery time of seat frame, which must be transported for *k*-th event counter,
- $f_{i}\left(k\right)$—is the forward transportation time between assembly stations for *k*-th event counter,
- $b_{i}\left(k\right)$—is the backward transportation time between assembly stations for *k*-th event counter.

**Figure 7:**
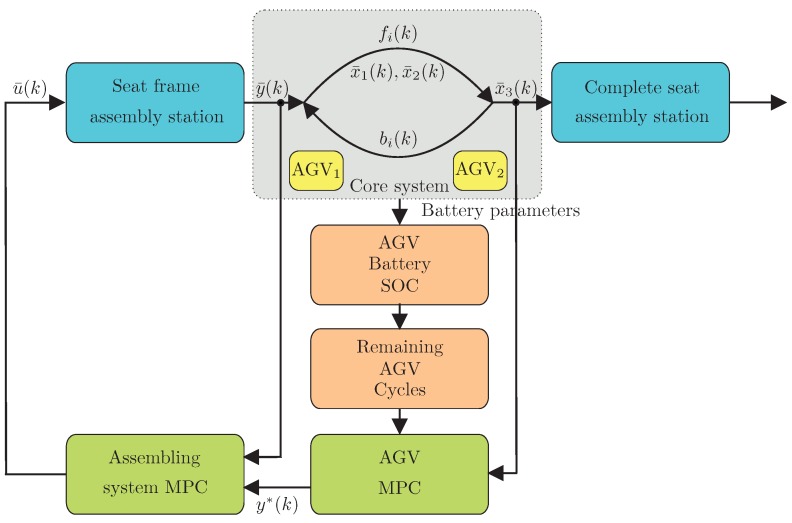
Sample transportation scenario: process.

The approach proposed in this paper is outlined by a flowchart presented in Figure 7 and structured as follows:

1. Assembly stations: seat frame and complete seat;
2. Core system: containing two cooperating AGVs. It consists of two AGVs, which can realize a single task. Thus, the problem is to spread the work load among these two cooperating AGVs in such a way to optimize their battery consumption process;
3. AGV-battery SOC: battery state estimator (Section 4);
4. Remaining AGV cycles: a state-of-charge prediction mechanism (Section 5).
5. AGV MPC: a health-aware predictive control (Section 7).
6. Assembling system MPC: the seat frame assembly station MPC.

It should be pointed out that *Assembling system MPC* employs the approach proposed in [60], and hence, its detailed description is omitted within the present paper.

As mentioned in Section 2, one important issue for efficient and effective AGV operation are SOC and SOH of the battery. This research aims to provide trustworthy estimates of these characteristics and allow predictive planning for optimum resource allocation in such assembly scenarios. Thus, with all the above variables, the task is to develop an algorithm able to calculate $\bar{y}\left(k\right)$ in such a way as to guarantee that ${\bar{x}}_{3}\left(k\right)$ will follow a predefined schedule $t_{ref}\left(k\right)$. The main assumption behind further developments is that a single AGV is not able to follow $t_{ref}\left(k\right)$, and hence, its cooperative functioning is required. As already mentioned, such a system can be understood as a one with concurrency (compare [71]). Finally, due to the relatively high weight of the seat and the long operation range (the path is longer than shown in Figure 5) it is desirable to be able to ensure a satisfactory battery SOC and SOH of the AGV. Thus, the main objective of this paper is to develop a model-predictive health-aware control for the cooperating AGVs. It results in a series of $y^{*}\left(k\right)$, which is used as output constraint $\bar{y}\left(k\right)$ of the seat frame assembly station (cf. Figure 7). As a result, its input $\bar{u}\left(k\right)$ is calculated according to the approach presented in [60], which is briefly outlined in Section 8.

The overall assembly station presented in Figure 5 is a DES. DES is defined as a discrete-state, event-driven system, while its state evolution depends solely on the occurrence of discrete events over time. It means that transitions are only observed at the discrete instants in time. There are many different modeling techniques for DESs, such as Petri nets, extended state machines, event-graphs, formal languages, generalized semi-Markov processes, nonlinear programming, automata, etc. However, most of them can be used as computer simulation models, and hence, their application to DES-control is usually limited.

One of the possible approaches, which can be used to settle this problem, is to employ a DES Model-Predictive Control (DESMPC) scheme developed by de Schutter and Van den Boom [72] for a special class of DES. This class can be modeled using the so-called max-plus algebra [64], i.e., an algebra involving two mathematical operators max(·) and +. The main advantage of DESMPC is associated with the fact that all variables involved in the system model represent occurrence times of particular events (e.g., AGV started transporting a seat), and hence, they are non-negative. This feature makes it possible to replace a quadratic programming problem (employed in a conventional MPC for continuous systems) by a linear programming one. It results in a significant reduction of computational burden. Despite the incontestable appeal of DESMPC, it does not allow coping with concurrency or health-awareness. Indeed, while considering two cooperating AGVs, a natural concurrency exists and only one of them can be selected to tackle the transportation task of a given seat. Subsequently, the MPC cost function is extended in such a way to cope with health-aware aspects related to the battery status of cooperating AGVs.

A detailed derivation of the novel strategy is presented in Section 7. However, its development requires appropriate measures of battery parameters. This problem is to be tackled in the subsequent sections.

## 4. Estimation of the Battery State

It should be noted that the model (3) is linear only with respect to state. The nonlinear output function g(·,·) described by Equation (4) causes the necessity of using nonlinear estimators such as EKF [34,48] to estimate the battery state and provide a novel approach for predicting its SOC and SOH. Despite the incontestable appeal of EKF, it may be divergent, and hence, the resulting estimates can be perceived as unreliable. Consequently, the entire control scheme will work with significantly degraded performance. It may even cause control action leading to the premature discharge of AGV batteries. Model (3)–(4) is further detailed by (7)–(8) along with a novel estimator structure, which overcomes the nonlinearity problem and takes into account inevitable disturbances/noise. Unlike the EKF, its convergence is proven by Theorem 2 in Section 4.

The battery SOC estimation is required in order to design the control system of the cooperative AGVs. This task is non-trivial due to the nonlinear nature of the processes taking place in the battery and described by (4). To solve such a problem, the EKF is usually considered (see [3], e.g., and the references therein). Despite the incontestable appeal of such an approach, it inherits a main disadvantage of EKF, which pertains its convergence. Thus, to settle this problem, a new estimator based on Luenberger observer which overcomes the nonlinearity problem and takes into account inevitable disturbances/noise will be developed in this part of the paper. In addition, the convergence of the proposed method will be proved using the Quadratic Boundedness (QB) approach [73]. In such an approach it is assumed that all uncertainties and in particular the estimation error can be limited by the ellipsoid. Such knowledge allows us to prove the convergence of the developed estimator.

Let us start by observing the fact that the output Equation (4) can be written as follows:

(6) $$\begin{array}{c} y_{l}=Cx_{l}+Du_{l}+h\left(x_{1,l}\right), \end{array}$$

where C=[0,1,1], $D=R_{0}$, $h\left(\cdot \right)=V_{OCV}\left(\cdot \right)$. It should be recalled that h(·) represents an average cell open circuit voltage. A practical way of determining this function is provided in the subsequent part of the text.

Finally, the above description can be extended in such a way to cope with measurement and process noise/disturbances:

(7) $$\begin{array}{cc} x_{l+1} & =Ax_{l}+Bu_{l}+W_{1}w_{l}, \end{array}$$

(8) $$\begin{array}{cc} y_{l} & =Cx_{l}+Du_{l}+h\left(x_{1,l}\right)+W_{2}v_{l}, \end{array}$$

where $w_{l}\in R^{n_{w}}$ and $v_{l}\in R$ stand for the process and output noise/disturbance, respectively. Taking into account the above observation, a novel estimator structure is proposed:

(9) $$\begin{array}{c} {\hat{x}}_{l+1}=A{\hat{x}}_{l}+Bu_{l}+K\left(y_{l}-C{\hat{x}}_{l}-Du_{l}-h\left({\hat{x}}_{1,l}\right)\right), \end{array}$$

where ${\hat{x}}_{l}$ is the state estimate while *K* stands for the estimator gain matrix. Please note that the structure of estimator (9) can be perceived as an extension of the conventional Luenberger observer for linear systems. Indeed, without $h({\hat{x}}_{1,l})$ it is compatible with it. Thus, by incorporating $h({\hat{x}}_{1,l})$ into (9), the proposed estimator can cope with the nonlinearities present in (8).

For further analysis, and in particular to prove the convergence of the developed estimator, let us define the state estimation error:

(10) $$\begin{array}{c} e_{l+1}=x_{l+1}-{\hat{x}}_{l+1}. \end{array}$$

Substituting (7)–(9) into (10) yields:

(11) $$\begin{array}{c} e_{l+1}=Ae_{l}+W_{1}w_{l}-K\left(Ce_{l}+h\left(x_{1,l}\right)-h\left({\hat{x}}_{1,l}\right)+W_{2}v_{l}\right). \end{array}$$

As it can be observed, the main difficulty with proving the convergence of the estimation error is due to the presence of nonlinearity:

(12) $$\begin{array}{c} h\left(x_{1,l}\right)-h\left({\hat{x}}_{1,l}\right). \end{array}$$

One way to settle this unappealing effect is to perform linearization as it is the case for EKF. However, this is the main factor contributing to the possible divergence of EKF. To avoid it, the following function is defined:

(13) $$\begin{array}{c} \gamma \left(x_{l},{\hat{x}}_{l}\right)=\left\{\begin{array}{cc} \frac{h\left(x_{1,l}\right)-h\left({\hat{x}}_{1,l}\right)}{x_{1,l}-{\hat{x}}_{1,l}} & x_{l}\neq {\hat{x}}_{l},\\ 0 & \mathrm{otherwise}. \end{array}\right. \end{array}$$

Please note that function (13) is simply a difference (12) divided by the state estimation error $e_{l}$. In case $e_{l}=0$ the division is not determined, and hence, $\gamma (x_{l},{\hat{x}}_{l})=0$. Introducing (13) into (11) boils down to:

(14) $$\begin{array}{c} e_{l+1}=Ae_{l}+W_{1}w_{l}-K\left(Ce_{l}+\gamma \left(x_{l},{\hat{x}}_{l}\right)e_{1,l}+W_{2}v_{l}\right). \end{array}$$

To proceed further, it is necessary to investigate the nature of $h_{x_{1,l}}$ (i.e., $V_{OCV}\left(SOC\right)$). Indeed, it can be described with two important observations:

- SOC is within the range $0<x_{1,l}<1$;
- the function h(·) linking SOC and $V_{OCV}$ is positive and monotonic.

These two important observations impose the following bounds on $\gamma (x_{l},{\hat{x}}_{l})$:

(15) $$\begin{array}{c} 0<\gamma \left(x_{l},{\hat{x}}_{l}\right)<\bar{\gamma }, \end{array}$$

with $\bar{\gamma }>0$ being a constant dependent on the shape of h(·), which allows rewriting (13) as:

(16) $$\begin{array}{c} \gamma \left(x_{l},{\hat{x}}_{l}\right)=\beta \bar{\gamma },\;\beta \in \left(0,1\right). \end{array}$$

Please note that $\bar{\gamma }>0$ can be assessed graphically by drawing (13) for $0<x_{1,l}<1$ and $0<{\hat{x}}_{1,l}<1$ and determining its maximum value. Furthermore, improvements in the estimation quality of $\bar{\gamma }$ can be obtained by using numerical routines for the constrained numerical optimization.

Subsequently, substituting (16) into (14) leads to

(17) $$\begin{array}{c} e_{l+1}=\left(A-K\bar{C}\left(\beta \right)\right)e_{l}+W_{1}w_{l}-KW_{2}v_{l}. \end{array}$$

where $\bar{C}\left(\beta \right)=\left[\beta \bar{\gamma },\;1,\;1\right]$. Finally, it can be written in the compact form:

(18) $$\begin{array}{c} e_{l+1}=X\left(\beta \right)e_{l}+Zz_{l}, \end{array}$$

where $z_{l}={\left[w_{l}^{T},v_{l}\right]}^{T}$ and:

(19) $$\begin{array}{c} X\left(\beta \right)=A-K\bar{C}\left(\beta \right),\;Z={\bar{W}}_{1}-K{\bar{W}}_{2},\;{\bar{W}}_{1}=W_{1}\left[I_{n_{w}}\;0_{n_{w}\times 1}\right],\;{\bar{W}}_{2}=W_{2}\left[0_{1\times n_{w}}\;1\right]. \end{array}$$

Note that the resulting system (18) is a forced dynamic system with the input $z_{l}$. This property excludes the possibility of a direct application of the Lyapunov theory for its convergence analysis. As a remedy, a QB approach is employed [73]. In contrast to the Lyapunov approach, it allows investigation of the stability of forced dynamic systems (18) under bounded $z_{l}$. Moreover, it introduces a design parameter, which allows optimization of the decay rate of the state estimation error $e_{l}$. This approach has already been proven to be useful for the development of the estimation and prediction schemes [12,74].

For further deliberations, it is assumed that the noise/disturbance vector $z_{l}$ is bounded as follows [73,74]:

(20) $$z_{l}\in E_{z},\;E_{z}=\left\{z:z^{T}Qz\leq 1\right\},\;Q≻0.$$

The matrix *Q* is determined by assuming the maximum values of the components of $z_{l}$, i.e., $|z_{i,l}|\leq {\bar{z}}_{i}$, =1,…,3. Let us remember that the first two represent process disturbance and modeling uncertainty while the last is associated with measurement noise. Thus, ${\bar{z}}_{3}$ can be determined relatively easily using the maximum bound of the battery voltage measurement quantization error. Setting of ${\bar{z}}_{1}$ and ${\bar{z}}_{2}$ is more sophisticated and depends on the batter model quality. Thus, by validating the model on a given data set one can determine them with sufficient quality. Finally, matrix *Q* shaping the ellipsoid (20) is determined as follows:

$$Q=\mathrm{diag}\left(\frac{1}{{\bar{z}}_{1}^{2}},\frac{1}{{\bar{z}}_{2}^{2}},\frac{1}{{\bar{z}}_{3}^{2}}\right).$$

The above deliberations allow introduction of QB paradigm. The subsequent Definition 1 [73,75,76] is adapted to the system (18) for which the Lyapunov function is defined by $V_{l}=e_{l}^{T}Pe_{l}$, P≻0:

**Definition** **1.** *The system *(18)* is strictly quadratically bounded for all allowable $z\in E_{z}$, if $V_{l}>1$ implies $V_{l+1}<V_{l}$ for any $z\in E_{z}$.*

It should be noted that the strict QB of (18) guarantees that $V_{k+1}<V_{l}$ for any $z\in E_{z}$ when $V_{l}>1$. The above assumption and definition allow formulating the main result of this section, which is given by the following theorem:

**Theorem** **1.** *The estimator *(9)* is convergent in the QB sense if there exists α∈(0,1), P≻0 and N such that the following inequality is satisfied for all β∈(0,1)*

(21) $$\begin{array}{c} \left[\begin{array}{ccc} -P-\alpha P & 0 & A^{T}P-\bar{C}{\left(\beta \right)}^{T}N^{T}\\ 0 & -\alpha Q & {\bar{W}}_{1}^{T}P-{\bar{W}}_{2}^{T}N^{T}\\ PA-N\bar{C}\left(\beta \right) & P{\bar{W}}_{1}-N{\bar{W}}_{2} & -P \end{array}\right]≺0. \end{array}$$

**Proof.**  Using *Definition* 1, according to [73] the system (18) is strictly QB if there exists P≻0 and a scalar α∈(0,1) such that:

(22) $$\begin{array}{c} \left[\begin{array}{cc} X{\left(\beta \right)}^{T}PX\left(\beta \right)-P+\alpha P & X{\left(\beta \right)}^{T}PZ\\ Z^{T}PX\left(\beta \right) & Z^{T}PZ-\alpha Q \end{array}\right]≺0, \end{array}$$

for all β∈(0,1). Thus, by using Schur complements to (22) and then multiplying both its sites by $\mathrm{diag}\left(I,I,P\right)$ and substituting:

(23) $$\begin{array}{c} N=PK, \end{array}$$

give (21), which completes the proof. □

Please note that (21) cannot be solved directly for any β∈(0,1). However, taking into account the linearity of $\bar{C}\left(\beta \right)$ with respect to β∈(0,1), two constants $\beta_{1}$ and $\beta_{2}$, which are sufficiently close to either 0 or 1, can be defined. It means that they should satisfy $\beta_{1}>0$ and $\beta_{2}<1$. Thus, solving (21) can be equivalently replaced by solving:

(24) $$\begin{array}{c} \left[\begin{array}{ccc} -P-\alpha P & 0 & A^{T}P-\bar{C}{\left(\beta_{i}\right)}^{T}N^{T}\\ 0 & -\alpha Q & {\bar{W}}_{1}^{T}P-{\bar{W}}_{2}^{T}N^{T}\\ PA-N\bar{C}\left(\beta_{i}\right) & P{\bar{W}}_{1}-N{\bar{W}}_{2} & -P \end{array}\right]≺0,\;i=1,2. \end{array}$$

Finally, the estimator design procedure can be realized as follows (20):

**Off-line:**
  1. Obtain the upper bound $\bar{\gamma }$,
  2. Select the overbounding matrix Q≻0 in (20),
  3. Select α∈(0,1), (20),
  4. Solve LMIs (24) and obtain the gain matrix $K=P^{-1}N$.
**On-line**
  1. Set ${\hat{x}}_{0}$ and l=0.
  2. Obtain the state estimate ${\hat{x}}_{l+1}$ according to (9).
  3. Set l=l+1 and go to Step 2.

Please note that as demonstrated in [74], α∈(0,1) can be used for controlling the convergence rate of the estimator, i.e., the larger α the faster the convergence. Thus, (24) should be solved along with iteratively modifying α∈(0,1). The procedure starts with setting α close to zero, solving (24). If (24) is solvable then α can be increased and the whole procedure is repeated. The parameter α can be also obtained in an optimal way by transforming (24) into Generalize Eigenvalue Optimization Problem (GEVP). However, the highest convergence rate is not necessarily associated with the best estimation quality, and hence, the above-mentioned iterative approach seems to be more suitable.

Note also that the off-line part of the algorithm is very simple and boils down to solving two linear matrix inequalities (24). This task can be easily realized by using, e.g., Yalmip along with MATLAB. In this case, (24) can be directly put into Yalmip solver. With the state estimate ${\hat{x}}_{k}$ and, in particular, ${\hat{x}}_{1,l}$ which is an estimate of the current SOC of the battery, it is possible to predict its behavior in the future. This task will be realized in the subsequent section.

## 5. Prognosis of the Remaining SOC

The remaining SOC of the battery associated with an AGV constitutes the main measure of its Health-Aware (H-A) performance defined in this paper. The main motivation behind such a reasoning is that each AGV battery should last for the entire production shift (usually 8–10 h). Indeed, their replacement or recharging during the production shift may have serious technical and economic consequences. Thus, the objective of the subsequent deliberations is to develop the predictor of remaining SOC. Since (7) is linear, a natural approach is to model it in a linear fashion:

(25) $$\begin{array}{c} x_{1,l,i}=a_{i}l\Delta T+d_{i},\;i=1,2, \end{array}$$

where $a_{i}$ and $d_{i}$ are unknown parameters shaping the relation between time lΔT and SOC $x_{1,l}$ for the battery of *i*th AGV. Please note that the actual SOC is not available, and hence (5) is replaced by:

(26) $$\begin{array}{c} {\hat{x}}_{1,l,i}=a_{i}l\Delta T+d_{i},\;i=1,2, \end{array}$$

where ${\hat{x}}_{1,l,i}$ is an estimate of SOC of *i*th AGV battery obtained with the estimator developed in the preceding section. To cope with the estimation problem of $a_{i}$ and $d_{i}$, a celebrated Recursive Least Square (RLS) algorithm is employed:

(27) $$\begin{array}{cc} {\hat{p}}_{l,i} & ={\hat{p}}_{l-1,i}+L_{l,i}\left({\hat{x}}_{1,l,i}-r_{l,i}^{T}{\hat{p}}_{l-1,1}\right), \end{array}$$

(28) $$\begin{array}{cc} K_{l,i} & =P_{l-1,i}r_{l,i}{\left(1+r_{l,i}^{T}P_{l-1,i}r_{l,i}\right)}^{-1}, \end{array}$$

(29) $$\begin{array}{cc} P_{l,i} & =\left[I_{2}-L_{l,i}r_{l,i}^{T}\right]P_{l-1,i}, \end{array}$$

where the parameter estimation vector along with its regressor are defined by:

(30) $$\begin{array}{c} {\hat{p}}_{l,i}={\left[{\hat{a}}_{l,i},\;{\hat{d}}_{l,i}\right]}^{T},\;r_{l,i}={\left[l\Delta T,\;1\right]}^{T}. \end{array}$$

To summarize, to obtain $a_{i}$ and $d_{i}$, the following algorithm is used:

- Set ${\hat{p}}_{0,i}={\left[0,\;1\right]}^{T}$, $P_{0,1}=\delta I_{2}$ and l=0, with δ>0 being a sufficiently large positive constant.
- Obtain parameter estimates using (27)–(29).
- Set l=l+1 and go to Step 2.

Please note that by observing (26) with the initial estimate ${\hat{p}}_{0,i}={\left[{\hat{a}}_{0,i},\;{\hat{d}}_{0,i}\right]}^{T}={\left[0,\;1\right]}^{T}$ it is evident that it corresponds to SOC equal one. Indeed, this is a natural approach to assume that the battery is fully loaded at the beginning of the operation of AGV.

Finally, by bridging the algorithm estimating SOC (presented in Section 4) along with the one estimating unknown parameters of (26), the following SOC predictor can be obtained

(31) $$\begin{array}{c} {\bar{x}}_{1,l_{x},i}={\hat{a}}_{i,l}l_{x}\Delta T+{\hat{d}}_{i,l},\;i=1,2, \end{array}$$

where $l_{x}$ is the discrete time of prediction which is based on the parameter estimates obtained up to *l*. It implies that $l_{x}\geq l$. The objective of the subsequent section is to evaluate the performance of the SOC estimation and prediction strategies.

## 6. Performance Evaluation

**Figure 8:**
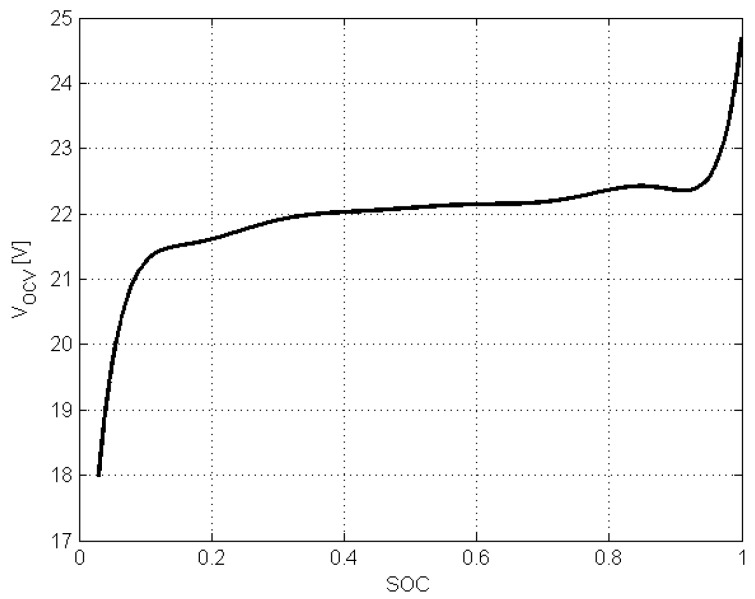
Function h(·).

**Figure 9:**
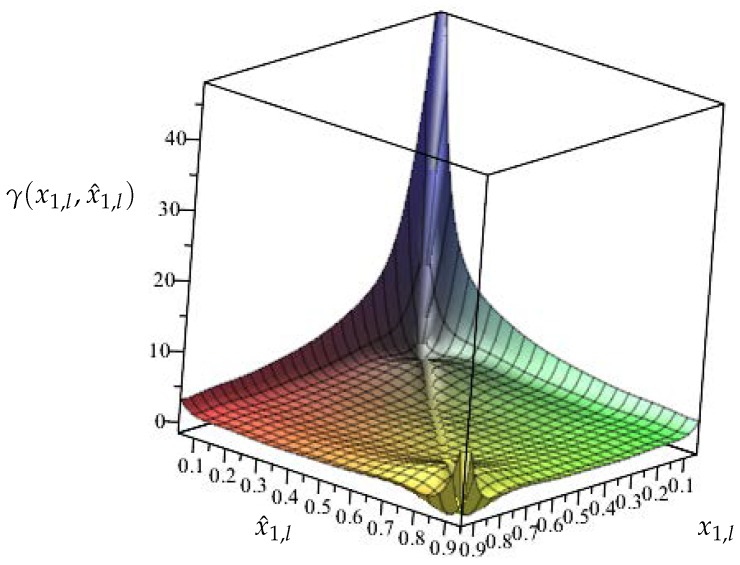
Function γ(·).

In current industrial applications, AGVs still use lead–acid batteries, mainly because of their low cost. However, main disadvantages are relatively large weight and volume for a desired capacity and long charging duration. Therefore, a battery with lithium-ion technology is assumed in this paper. The battery uses a special kind of lithium-Ion technology—the lithium nickel manganese cobalt oxide technology (Li-NMC). This technology is also used in modern cars and excels with high energy density and low self-heating. The cell voltage is between 3.6 and 3.7 V. The battery pack, which is considered for both AGVs, has a nominal voltage of 25.2 V and a nominal capacity of 200 Ah. Let us start by providing numerical parameters related to the battery description (3) and (4): $Q_{nom}=200$ Ah, $R_{CT}=0.051$ Ω, $C_{CT}=0.1922$ F, $R_{Dif}=0.0126$ Ω, $C_{Dif}=0.8213$ F, $R_{0}=0.05$ Ω, Δt=0.1 s. With the above parameters, the preliminary step is to determine h(·) of the battery pack. For that purpose, the general approach proposed in [77] along with a suitable polynomial regression function were employed. Figure 8 and Figure 9 present functions h(·) and γ(·).

**Figure 10:**
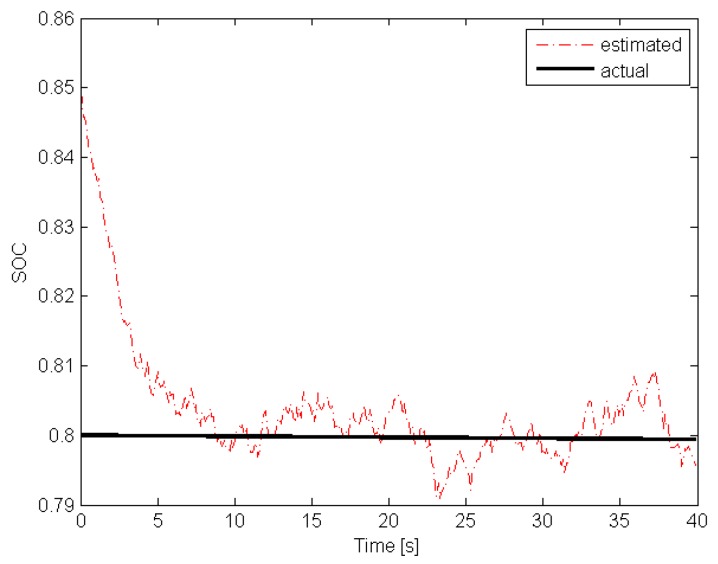
Actual SOC and its estimate (initial estimation phase).

**Figure 11:**
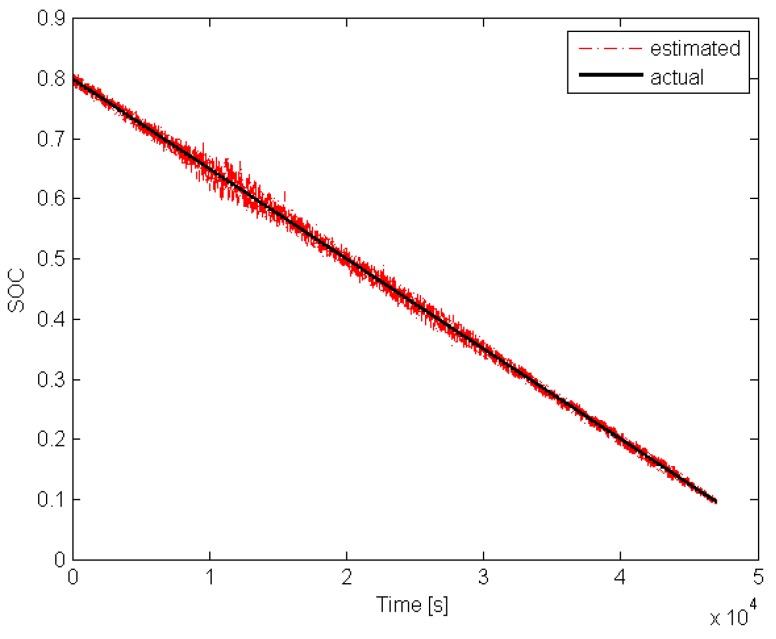
Actual SOC and its estimate.

With the above preliminary information, it is possible to start *off-line* phase of the algorithm proposed in Section 4. Indeed, the upper bound of γ(·) can be easily determined and it is equal to $\bar{\gamma }=45$. The next step is to select matrix Q>0 shaping the overbounding ellipsoid of $w_{k}$ and $v_{k}$. Taking into account the quantization error of the measurement unit along with possible changes of the internal capacities/resistances of the battery, it was selected as $Q=10I_{n_{w}+1}$. The next step is to select the parameter α∈(0,1) shaping the convergence of the estimator. The highest possible value was selected α=0.99 and as a result of solving LMIs (24) the estimator gain matrix was obtained $K={\left[0.0225,0.0001,0.0011\right]}^{T}$. With the above parameters, the initial estimate was set to ${\hat{x}}_{0}={\left[0.85,0,0\right]}^{T}$. The investigation resembled a usual duty cycle of AGV consisting of driving and stopping phases without an additional load. It is important to underline that no alternate discharge/charge was performed, which could be a possible cause of an error in the actual SOC prediction. The total investigation covered a usual two-shift timespan. The results that were achieved during the whole cycle are presented in Figure 10 and Figure 11.

Please note that the initial actual SOC was at the level of 0.8 while ${\hat{x}}_{1,0}=0.85$ was selected intentionally to exhibit the convergence of the proposed estimator. The obtained results clearly show that the estimated SOC converges towards the actual one within an acceptable time and tracks it accurately enough during the entire investigation.

**Figure 12:**
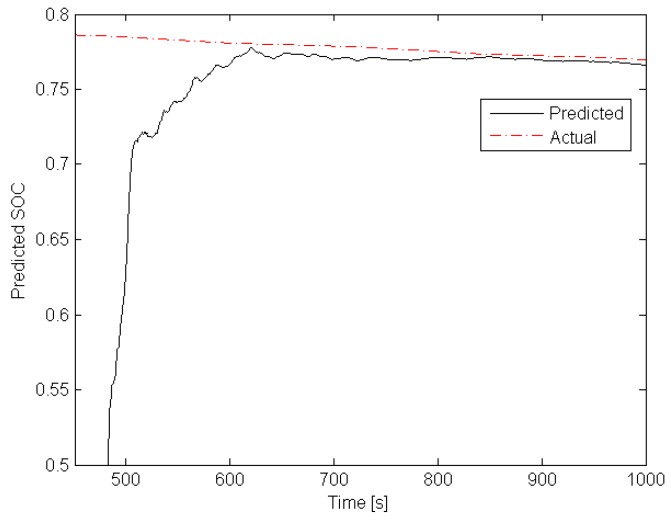
SOC and its prediction—initial prediction phase.

**Figure 13:**
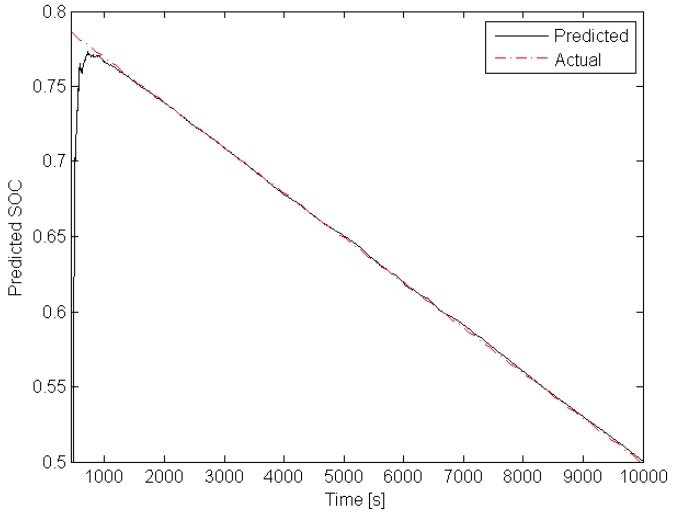
SOC and its prediction.

The above result recommends using the obtained estimator for predicting SOC according to the approach proposed in Section 5. For this scenario, a typical AGV working sequence was generated, which consists of two alternate phases: 2 min drive with load and 2 min drive without load. Please note that due to the load, the average current is typically increasing of ca. 47%. The task was to predict SOC after 7.5 min from each *l*. This means $l_{x}$ in (31) was set to $l_{x}=l+4500$. Figure 12 and Figure 13 present the obtained predictions along with their actual values. These results clearly indicate that the uncertainty of the initial SOC value, which was set to 0.85 instead of actual one equal 0.8, causes a large prediction error in the initial phase. Such a behavior can also be deduced from Figure 10.

Note that the time axis varies from 0 to 10,000 s (determined by $b_{i}$ and $f_{i}$), but the load is alternately changed every 120 s. Indeed, each loaded AGV and empty AGV phases last 2 min. However, after a transient phase, the prediction quality performance increases significantly. The above results recommend applying the proposed SOC estimation/prediction scheme within each of redundant AGVs. This is the objective of the subsequent part of this paper.

## 7. Predictive Control of the Assembly System

The result of earlier research work is an MPC-based fault-tolerant strategy (compare Section 2). This section introduces this kind of strategy for a system with redundancy and under consideration of SOC and SOH of AGV batteries. Firstly, the general framework is explained; the battery aspects are considered in Section 7.2.

### 7.1. MPC of Cooperative AGVs

In this paper, it is proposed to describe a system with redundancy/concurrency presented in Figure 7 in the following way:

(32) $$\begin{array}{c} {\bar{x}}_{1}\left(k+1\right)=\max\left({\bar{x}}_{1}\left(k\right)+f_{1}\left(k\right)+b_{1}\left(k\right),y\left(k+1\right)+v_{1}\left(k+1\right)\right),\\ {\bar{x}}_{2}\left(k+1\right)=\max\left({\bar{x}}_{2}\left(k\right)+f_{2}\left(k\right)+b_{2}\left(k\right),y\left(k+1\right)+v_{2}\left(k+1\right)\right),\\ {\bar{x}}_{3}\left(k+1\right)=\max({\bar{x}}_{1}\left(k+1\right)+f_{1}\left(k+1\right)+v_{1}\left(k+1\right),{\bar{x}}_{2}\left(k+1\right)+f_{2}\left(k+1\right)+v_{2}\left(k+1\right). \end{array}$$

where $v_{1}\left(k\right),v_{2}\left(k\right)\in \left\{0,\varepsilon \right\}$, ε=−inf, are decision variables taking the value either 0 or −inf. It goes without saying that $v_{1}\left(k\right)$ and $v_{2}\left(k\right)$ cannot have identical value as *i*th AGV performing transport for *k*th event counter has $v_{i}\left(k\right)=0$ while the remaining one has $v_{j}\left(k\right)=\varepsilon$. Such a description requires additional constraint of the form:

(33) $$\begin{array}{c} \mathrm{If}\;v_{i}\left(k\right)=\varepsilon ,\;\mathrm{then}\;f_{i}\left(k\right)=0,\;\;b_{i}\left(k\right)=0, \end{array}$$

which simply means if *i*th AGV does not perform transportation task then its forward and backward time are equal to zero. With the model of the cooperative AGVs described by (32) the problem is to find values of switching variables $v_{i}\left(k\right)$ in such a way as to maximize:

(34) $$J_{y}=\sum_{k=k_{0}}^{k_{0}+N_{p}}\bar{y}\left(k\right),$$

starting from an initial event counter $k_{0}$ on a given prediction horizon $N_{p}$ in such a way as to achieve a desired schedule ${\bar{x}}_{3}\left(k\right)\leq t_{ref}\left(k\right)$.

Despite the incontestable appeal and relative simplicity of the above description (32)–(34), it cannot be solved directly. Thus, the objective of the subsequent part of this section is to transform the above optimization task into Mixed-integer Linear Programming (MLP) [78].

Thus, let us start with describing possible decision situations with a binary variable z(k):

(35) $$z\left(k\right)=\left\{\begin{array}{cc} 0 & v_{1}\left(k\right)=\varepsilon \;\mathrm{and}\;v_{2}\left(k\right)=0,\\ 1 & v_{1}\left(k\right)=0\;\mathrm{and}\;v_{2}\left(k\right)=\epsilon , \end{array}\right.$$

with such a binary variable, an application of an operator max(·) in (32) can be replaced by a set of equivalent constraints for the first:

(36) $$\begin{array}{c} {\bar{x}}_{1}\left(k+1\right)\geq {\bar{x}}_{1}\left(k\right)+z\left(k\right)\left(f_{1}\left(k\right)+b_{1}\left(k\right)\right),\\ {\bar{x}}_{1}\left(k+1\right)\geq \bar{y}\left(k+1\right)+\left(1-z\left(k+1\right)\right)\varepsilon , \end{array}$$

and second:

(37) $$\begin{array}{c} {\bar{x}}_{2}\left(k+1\right)\geq {\bar{x}}_{2}\left(k\right)+\left(1-z\left(k\right)\right)\left(f_{2}\left(k\right)+b_{2}\left(k\right)\right),\\ {\bar{x}}_{2}\left(k+1\right)\geq \bar{y}\left(k+1\right)+\left(1-z\left(k+1\right)\right)\varepsilon . \end{array}$$

AGV, respectively. While, the seat frame arrival time ${\bar{x}}_{3}\left(k\right)$ obeys:

(38) $$\begin{array}{c} {\bar{x}}_{3}\left(k+1\right)\geq {\bar{x}}_{1}\left(k+1\right)+f_{1}\left(k+1\right)+\left(1-z\left(k+1\right)\right)\varepsilon ,\\ {\bar{x}}_{3}\left(k+1\right)\geq {\bar{x}}_{2}\left(k+1\right)+f_{2}\left(k+1\right)+z\left(k+1\right)\varepsilon . \end{array}$$

Before proceeding to the development of the predictive control algorithm let us formally define the remaining constraints:

- first, the designed system must follow some predefined trajectory that can be defined as scheduling constraints of the form:
  (39) $$\begin{array}{c} {\bar{x}}_{3}\left(k\right)\leq t_{\mathrm{ref}}\left(k\right), \end{array}$$
  where $t_{ref}\left(k\right)$ is the upper bound of ${\bar{x}}_{3}\left(k\right)$ at *k*.
- the second set of constraint concerns the performance of the AGVs:
  (40) $$\begin{array}{c} {\bar{x}}_{i}\left(k+1\right)-{\bar{x}}_{i}\left(k\right)\leq {\bar{f}}_{i}\left(k\right),\;i=1,2. \end{array}$$
  The possibly time-varying upper bounds ${\bar{x}}_{i}\left(k\right)$ is connected to the minimum speed of the AGVs.
- the last constraint is the rate of change one:
  (41) $$\begin{array}{c} {\bar{x}}_{i}\left(k+1\right)-{\bar{x}}_{i}\left(k\right)\geq c_{i},\;i=1,2. \end{array}$$
  where $c_{i}>0$ is lower bound of the change rate.

Obviously, the time of reaching the individual station for k+1 cannot be smaller than the one for *k*. Using constraints (36)–(41), an MPC strategy can be developed.

In the first stage the following cost function is introduced:

(42) $$J_{y}=\sum_{k=k_{0}}^{k_{0}+N_{p}}\bar{y}\left(k\right)$$

and, hence the optimization task is:

(43) $$\left(y^{*}\left(k_{0}\right),\cdots y^{*}\left(k_{0}+N_{p}\right)\right)=\arg\;\max_{\underset{z\left(k_{0}\right),\cdots ,z\left(k_{0}+N_{p}\right)}{\underset{\bar{y}\left(k_{0}\right),\cdots ,\bar{y}\left(k_{0}+N_{p}\right)}{\bar{x}\left(k_{0}\right),\cdots ,\bar{x}\left(k_{0}+N_{p}\right)}}}J_{y},$$

under (36)–(41). Finally, it should be pointed out that the above optimization problem is a mixed-integer linear programming problem, which can be solved with a wide spectrum of computational packages, e.g., MATLAB.

The above predictive strategy is used sequentially to obtain $y^{*}\left(k_{0}\right)$ for all $k_{0}=0,1,2,\ldots$, while the resulting $y^{*}\left(k_{0}\right)$ is used as a scheduling constraint $\bar{y}\left(k_{0}\right)\leq y^{*}\left(k_{0}\right)$ for the seat frame assembly system portrayed in Figure 7. As a result, its optimal predictive control $\bar{u}\left(k_{0}\right)$ can be computed using one of the strategies proposed in [60]. The latter task is however beyond the scope of this paper. Note that the above strategy does not take into account the current SOH of AGV batteries, and hence, it cannot guarantee its balanced exploitation. Indeed, the cost function (42) clearly aims at finding the slowest possible production scenario satisfying a predefined schedule.

### 7.2. Health-Aware MPC of Cooperative AGVs

**Figure 14:**
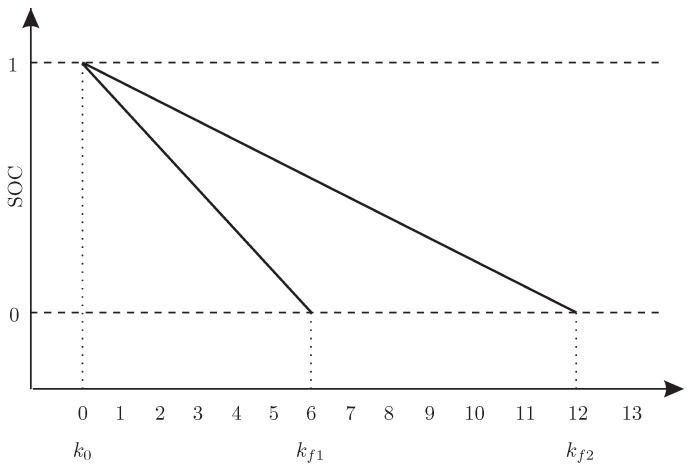
Illustration of the state of health of batteries.

Let us start with reminding the definition stated in the introductory part of the paper: the SOH is several cycles $k_{f}$ from a given SOC down to zero. This definition is illustrated in Figure 14 where $k_{f,1}$ represents the feasible number of cycles of $AGV_{1}$ while $k_{f,2}$ stands for an analogous variable associated with $AGV_{2}$.

Thus, the SOH of $AGV_{1}$ is definitely lower than the one of $AGV_{2}$. From SOC prediction Equation (31), it can be deduced that by substituting $l_{x}\Delta t=k_{f,i}(f_{i}\left(k_{0}\right)+b_{i}\left(k_{0}\right)+l\Delta t$, the number of cycles $k_{f,i}$ to zero SOC obeys:

(44) $$0={\hat{a}}_{i,l}(k_{f,i}\left(f_{i}\left(k_{0}\right)+b_{i}\left(k_{0}\right)+l\Delta t\right)+{\hat{d}}_{i,l}.$$

Please note that the forward and backward drive time $f_{i}\left(k_{0}\right)+b_{i}\left(k_{0}\right)$ is used for the prediction purpose. It means that this it is assumed constant while making the above prediction, i.e., $f_{i}\left(k\right)+b_{i}\left(k\right)=f_{i}\left(k_{0}\right)+b_{i}\left(k_{0}\right)$, for $k=k_{0},\ldots ,k_{0}+N_{p}$.

Finally, the total number of forward and backward cycles of *i*th AGV can be estimated as:

(45) $$k_{f,i}=\left\lfloor -\frac{{\hat{a}}_{i,l}l\Delta t+{\hat{d}}_{i,l}}{{\hat{a}}_{i}\left(f_{i}\left(k_{0}\right)+b_{i}\left(k_{0}\right)\right)}\right\rfloor ,$$

where ⌊·⌋ rounds the resulting value to the smallest positive integer.

Thus, for a given $k_{0}$ the remaining number of cycles is equal to:

(46) $$k_{r,i}=k_{f,1}-k_{0}.$$

With the above result, for each AGV a H-A cost function is introduced:

(47) $$J_{h,1}=\left(f_{1}\left(k_{0}\right)+b_{1}\left(k_{0}\right)\right)k_{r,1}-\left(f_{1}\left(k_{0}\right)+b_{1}\left(k_{0}\right)\right)\sum_{k=k_{0}}^{k_{0}+N_{p}}\left(1-z\left(k\right)\right),$$

where $\left(f_{1}\left(k_{0}\right)+b_{1}\left(k_{0}\right)\right)k_{r,1}$ stands for the total operational time of first AGV, while $\left(f_{1}\left(k_{0}\right)+b_{1}\left(k_{0}\right)\right)\sum_{k=k_{0}}^{N_{p}}\left(1-z\left(k\right)\right)$ stands for its exhausted portion:

(48) $$J_{h,2}=\left(f_{2}\left(k_{0}\right)+b_{2}\left(k_{0}\right)\right)k_{r,2}-\left(f_{2}\left(k_{0}\right)+b_{2}\left(k_{0}\right)\right)\sum_{k=k_{0}}^{k_{0}+N_{p}}z\left(k\right).$$

As a result, the total H-A cost function of cooperating AGV, which must be maximized, is:

(49) $$J_{h}=J_{h,1}+J_{h,2}.$$

Finally, the cost function $J_{y}$ (42) can be merged with $J_{h}$ in the following way:

(50) $$J=\left(1-\kappa \right)J_{y}+\kappa J_{h},$$

where 0≤κ≤1.

Thus, when κ=0 then the cost function does not take into account the SOC and SOH of the batteries in the two AGVs. In contrast, when κ=1 then the SOC and the SOH are the only important optimization ingredients. Thus, the designer must find an appropriate trade-off between these two situations by the appropriate selection of κ.

Finally, the developed H-A control strategy involves two algorithms running in parallel, i.e., SOC prognosis resulting in current values of ${\hat{a}}_{i,l}$ and ${\hat{d}}_{i,l}$ (cf. Section 5) and the predictive control being proposed in this section, which can be summarized as follows:

**Step 0:** Set $N_{p}>0$, κ∈[0,1], x(0), k=0.
**Step 1:** Set $k_{0}=k$ and calculate $k_{f,1}$ and $k_{f,2}$ according to (45).
**Step 2:** Solve a mixed-integer linear programming problem
  (51) $$\left(y^{*}\left(k_{0}\right),\cdots y^{*}\left(k_{0}+N_{p}\right)\right)=\arg\;\max_{\underset{z\left(k_{0}\right),\cdots ,z\left(k_{0}+N_{p}\right)}{\underset{\bar{y}\left(k_{0}\right),\cdots ,\bar{y}\left(k_{0}+N_{p}\right)}{\bar{x}\left(k_{0}\right),\cdots ,\bar{x}\left(k_{0}+N_{p}\right)}}}J,$$
  under (36)–(41).
**Step 3:** Apply $\bar{y}\left(k\right)=y^{*}\left(k_{0}\right)$ to the cooperative AGV system with $z(k_{0})$ determining AGV performing transportation for *k*th event counter.
**Step 4:** Set k=k+1 and go to *Step 1*.

## 8. Verification and Experimental Results

The objective of this section is to perform a comprehensive performance analysis of the H-A MPC algorithm proposed in the preceding section using 3 different scenarios:

**Scenario 1:**κ=0—predictive control without H-A features.
**Scenario 2:**κ=0.5—predictive control with H-A features, AGV batteries with equal SOC.
**Scenario 3:**κ=0.5—predictive control with H-A features, SOC of $AGV_{1}$ smaller than SOC of $AGV_{2}$.

The investigations were realized with the actual measurements taken from the AGV intended to be working within the cooperating AGV system. This system is structured as a proof of concept, which means that it mimics the prospective industrial system for which the proposed algorithm can be implemented.

For all scenarios, the initial condition x(0)=0 was used while the scheduling constraint $t_{ref}\left(k\right)$ was shaped by the following sequence:

(52) $$\begin{array}{c} 10,\;13,\;15,\;18,\;21,\;23,\;26,\;29,\;31,\;34,\;37,\;39,\;\ldots \end{array}$$

The irregularity of the above sequence was introduced intentionally in such a way as to avoid a simple alternate switching between two cooperative AGVs.

**Figure 15:**
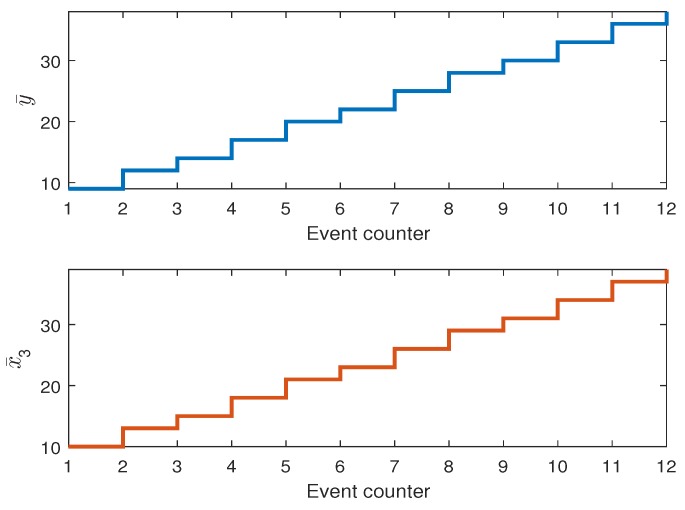
Scenario 1: Optimal sequence $\bar{y}\left(k\right)$ for AGVs.

**Figure 16:**
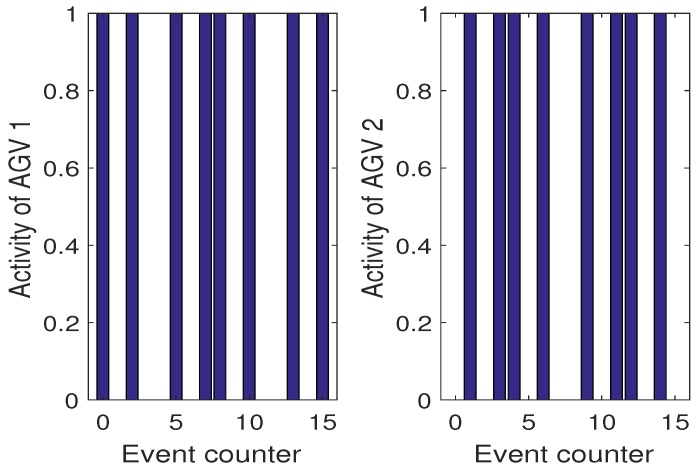
Scenario 1: Activities of AGVs.

Figure 15 shows the optimal sequence of $\bar{y}\left(k\right)$ and ${\bar{x}}_{3}\left(k\right)$ satisfying (52) obtained for Scenario 1. Please note that in this scenario no H-A features are considered. However, as it can be observed in Figure 16 the activities of two collaborative AGVs are equally spread during the operational time.

**Figure 17:**
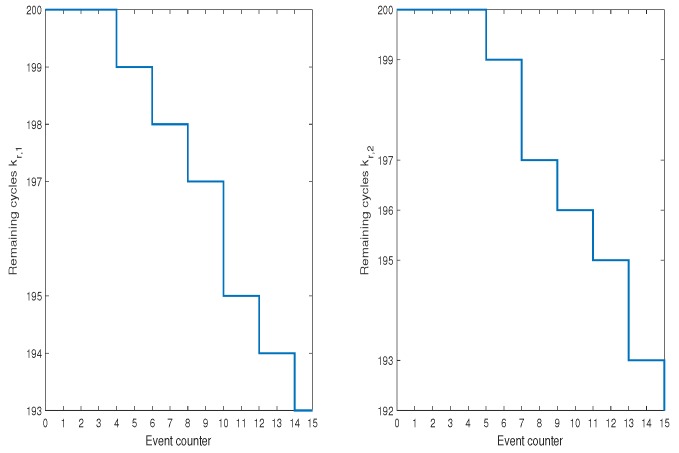
Scenario 2: SOH of AGVs expressed by the remaining cycles $k_{f,i}$.

**Figure 18:**
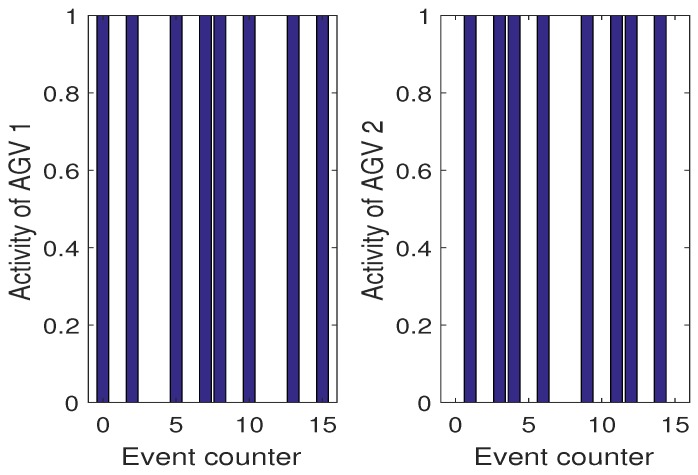
Scenario 2: Activities of AGVs.

However, one can imagine a less demanding schedule than (52) for which such a balance will be lost in favor of more aggressive usage of one AGV only. In this case, it is possible that one algorithm will lose its SOH completely while the other one will expose a full one. This situation can be avoided by applying the proposed approach but first let us consider Scenario 2 with H-A features but the same SOH expressed by their respective $k_{f,i}$. First, it should be pointed out that the optimal $\bar{y}\left(k\right)$ and resulting ${\bar{x}}_{3}\left(k\right)$ are obtained the same for all scenarios while the difference lies in way of using AGVs. In Scenario 2, the activity of each AGV is the same as in Scenario 1. Figure 17 also shows the evolution of $k_{f,i}$ from which it can be observed that they are decreasing uniformly. In this case, the activities of both AGVs are shown in Figure 18.

**Figure 19:**
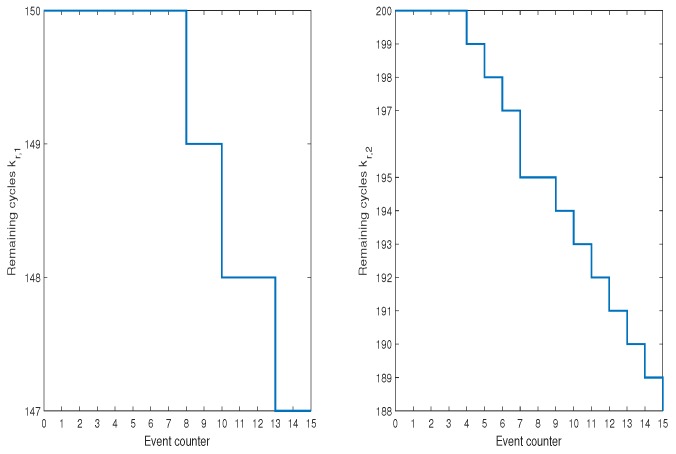
Scenario 3: SOH of AGVs expressed by the remaining cycles $k_{f,i}$.

**Figure 20:**
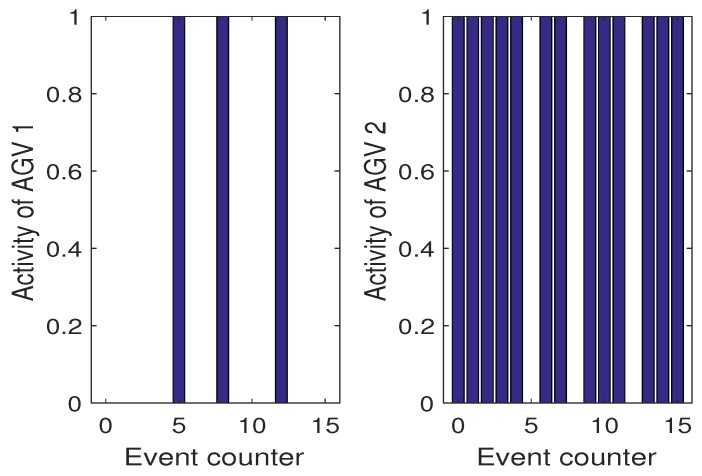
Scenario 3: Activities of AGVs.

However, it is enough to have one AGV with smaller $k_{f,i}$ to see immediately the profits that can be gained while using the proposed algorithm. Thus, let us consider Scenario 3 which exhibits such a situation. Figure 19 shows the evolution of respective $k_{f,i}$. From this result as well from Figure 20 it is an obvious fact that $AGV_{2}$ is more exploited while $AGV_{1}$ is used only while it is necessary. These results clearly prove that the proposed algorithm allows balanced exploitation of the collaborative AGV system, which undoubtedly increases their operational abilities.

## 9. Summary and Outlook

The automotive industry is currently undergoing a profound change because of electro-mobility and autonomous driving. This has lead to enormous challenges for this industry branch and especially to a need for increased flexibility. AGVs in production systems are one means for increasing flexibility but require elaborating H-A control systems. This paper presents a novel approach to include considerations of the SOC and the SOH into planning and control of DESs with AGVs. The outcomes of the paper can be summarized as follows:

**Novel AGV-battery SOC estimation strategy with guaranteed convergence:** in contrast to the approaches presented in the literature, the main attention was on guaranteeing the convergence of SOC estimation. The schemes presented in the literature either ignore the nonlinearity of the battery model or employ time-varying linearization paradigms such as EKF. It may lead to a larger estimation inaccuracy or even to the divergence of the estimates. Such a behavior does not recommend using such schemes for the H-A control purposes. Indeed, one can easily imaging a situation in which unreliable H-A AGV control may lead to the total inability of performing AGV mission due to the total and unpredicted discharge of its battery. The proposed estimator is guaranteed to converge, and hence, it can be perceived as a reliable source of current SOC. Such a performance was indicated during the experimental study.

**SOC prognosis scheme:** The battery SOC estimation scheme allows determination of its current value. However, such information is insufficient from the viewpoint of H-A control. Indeed, it requires information about SOC expected value over future time. Owing to the assumption about linear SOC evolution, a linear prediction model was assumed, which is accompanied with a linear RLS-based estimation scheme. Such a strategy allows continuous updating of the predicted SOC. The main advantages of the proposed approach are that it is based on reliable SOC estimates and its practical implementation reduces to the celebrated RLS algorithm. This aspect is very important from the viewpoint of prospective implementations on embedded AGV platforms. Finally, the performance of the SOC prediction strategy was clearly shown during performing series of experiments, which clearly exhibit its high prediction abilities.

**State-of-health-based scheme:** Current SOC estimates and its predicted values are very important measures of AGV possible performance. However, they do not directly translate into practical information related to the possible forward-backward transportation cycles of a given AGV. Thus, the main objective was to develop a scheme translating SOC and its predicted values into the remaining number of forward-backward transportation cycles. As a result, a practical measure describing defined as a SOH of a given AGV was obtained, which can be easily interpreted by the human operator as well as fed into H-A DESMPC.

**Health-aware DESMPC for cooperating AGVs:** The development concerning H-A DESMPC was oriented towards answering two important problems: How to introduce H-A information into DESMPC, and how to cope with concurrency of two cooperating AGVs within DESMPC. Within the authors’ knowledge, these questions remain unsolved before the developments presented in this paper. In particular, the answer to the former question was provided by developing a H-A cost function incorporating both AGV performance requirements along with their H-A capabilities. The answer to the latter problem was provided by introducing two decision variables along with their appropriate introductions into the cooperating AGVs model. Apart from the above-listed tasks, the overall optimized H-A control problem was accompanied with a set of constraints assuring its feasibility. Finally, the overall optimization problem was formulated into a mixed-integer linear programming one. This clearly indicated that the proposed approach does not involve a large computational burden. This clearly recommends its software implementation on various industrial platforms.

Thus, a general objective of the presented framework was to balance the SOH of the batteries in the system, which leads to uncompromised operation possibilities. The resulting framework for the H-A MPC was applied to a scenario with cooperative, redundant AGVs, and clearly indicated its application potential. For reasons of simplicity, no recharge scenarios have been considered in the presented research—it is one interesting future research direction. However, in most cases, AGV batteries are simply replaced after the shift while the discharged ones undergo the charging process.

Future research directions would include shared resources in the assembly scenario and would also aim at the fault-tolerant control. The latter can cope with AGV delays or even breakdown, which is very important from an industrial reality viewpoint.
